# Robotic-Assisted Redo Coronary Artery Bypass Grafting: A Single-Center Experience

**DOI:** 10.5761/atcs.oa.25-00026

**Published:** 2025-04-23

**Authors:** Yoshiyuki Yamashita, Gianluca Torregrossa, Serge Sicouri, Mary Ann C. Wertan, Danielle D Spragan, Basel Ramlawi, Francis P Sutter

**Affiliations:** 1 Department of Cardiac Surgery, Lankenau Heart Institute, Main Line Health, Wynnewood, PA, USA; 2 Department of Cardiothoracic Surgery Research, Lankenau Institute for Medical Research, Wynnewood, PA, USA

**Keywords:** coronary artery disease, Da Vinci surgery, minimally invasive cardiac surgery, off-pump coronary artery bypass grafting

## Abstract

**Purpose:** To report our experience with robotic-assisted redo coronary artery bypass grafting (CABG).

**Methods:** This single-center retrospective study included patients undergoing robotic-assisted redo CABG between 2016 and 2023. Patient demographics and operative outcomes were compared with those of initial robotic-assisted CABG procedures performed during the same period.

**Results:** There were 12 patients undergoing robotic-assisted redo CABG, with a median age of 73 years. Compared to initial CABG patients (n = 1415), the Society of Thoracic Surgeons scores were significantly higher (median: 0.90 vs. 7.05, p <0.001) in the redo group. Six patients had de novo internal mammary artery (IMA) to left anterior descending (LAD) bypass, 4 had redo LAD bypass, and 2 had non-LAD bypass. Among the 10 patients with LAD bypass, 4 also underwent hybrid percutaneous coronary intervention. While operating room time (5.4 vs. 7.4 hours, p <0.001), postoperative lengths of stay (4.0 vs. 5.5 days, p = 0.02) and the need for blood transfusion (15% vs. 42%, p = 0.02) were significantly greater in the redo group compared to the initial group, there were no conversions to sternotomy, unplanned revascularization, or in-hospital mortality in the redo patients.

**Conclusion:** Robotic-assisted redo CABG demonstrated promising operative outcomes in appropriately selected patients despite the higher-risk cohort.

## Introduction

Coronary artery bypass grafting (CABG) is the standard of care for revascularization of complex coronary artery disease and provides long-term survival benefits for patients with coronary artery disease.^1,2)^ Nonetheless, there are limitations in bypass graft patency, with reported 10-year graft patency rates of approximately 85%–95% for internal mammary artery (IMA) grafts and 60% for saphenous vein grafts (SVGs).^3,4)^ Despite optimal medical therapy, patients with previous CABG sometimes require additional coronary interventions due to bypass graft failure or disease progression in native coronary vessels. Both percutaneous coronary intervention (PCI) and redo CABG in patients with prior CABG are associated with higher risks of procedural failure and complications compared with those in patients without prior CABG,^5,6)^ and the optimal revascularization strategies for patients with prior CABG remain controversial. According to the 2021 American College of Cardiology/American Heart Association/Society for Cardiovascular Angiography and Interventions guidelines,^6)^ various factors influence the choice of revascularization modality, including the availability of the IMA, a patent graft to the left anterior descending artery (LAD), the anatomic complexity of the native and graft disease, and the feasibility of the revascularization method in addition to patient factors. These guidelines emphasize the importance of a heart team approach and shared decision-making. For example, in patients with a patent IMA-LAD graft, PCI for non-LAD lesions is recommended over CABG, whereas in patients with refractory angina due to LAD disease, it is reasonable to choose CABG over PCI if IMA is available for LAD bypass (both Class 2a recommendations). However, PCI is not always suitable for revascularization, and conventional CABG is an invasive procedure that typically involves sternotomy and the use of cardiopulmonary bypass, which carries an increased risk of operative mortality and morbidity, especially in the redo case.^7,8)^ To meet the demand for less invasive surgical procedures, minimally invasive techniques for CABG have been developed, including robotic-assisted CABG. This procedure typically consists of robotic harvesting of the left IMA (LIMA), followed by off-pump direct anastomosis of the LIMA to LAD through a left mini-thoracotomy. This approach offers several advantages over the traditional sternotomy approach, including a reduced need for blood transfusions, lower rates of surgical site infection, and faster recovery time while maintaining clinical outcomes comparable to traditional CABG.^9–12)^ In redo cases, this technique is potentially beneficial to avoid the risk of redo sternotomy. However, some institutions have considered robotic-assisted CABG contraindicated in patients with prior sternotomy,^13)^ and the efficacy of robotic-assisted redo CABG is poorly reported. We hypothesize that robotic-assisted redo CABG is a feasible and effective option in appropriately selected patients. The aim of this study is to report our experience with this procedure.

## Materials and Methods

### Patients and surgical technique

We retrospectively reviewed data on patients who underwent redo robotic-assisted CABG at a single institution in the United States between January 2016 and December 2023. The demographics and operative outcomes of these patients were compared with those who underwent initial robotic-assisted CABG during the same time period. Patients with prior cardiac surgery other than CABG were excluded from the initial robotic-assisted CABG group. The study protocol was approved by our Institutional Review Board (IRB 45CFR164.512). Individual patient consent was waived due to the retrospective nature of the study.

The detailed technique of robotic-assisted CABG has been described previously.^14–16)^ It is performed under general anesthesia with the insertion of a bronchial blocker, which allows single-lung ventilation. In general, the bypass procedure involves robotic harvesting of the LIMA in a skeletonized fashion, followed by off-pump direct anastomosis to the LAD through a small left anterior thoracotomy. The robotic procedures begin with the insertion of 3 ports, usually in the left midclavicular/anterior axillary line in the 2nd, 4th, and 6th intercostal spaces. The 3-dimensional view provided by the robotic platform enhances visualization of the LIMA. The right IMA (RIMA) can also be harvested using this approach. For redo cases, the robotic procedure includes dissection of adhesions and pericardium, identification of the target vessel, and harvesting of the IMA. If previous bypass grafts are present, they are also identified during the procedure. After harvesting the IMA, an off-pump direct coronary anastomosis is performed by extending the incision of the camera port site. When a free graft is used, one end of the graft is connected to the in situ IMA in a Y-shaped configuration, while the other end is anastomosed to target vessels accessible via thoracotomy, such as diagonal and ramus intermedius branches.

Robotic-assisted CABG is primarily indicated for patients with severe ostial LAD disease, long LAD lesions that are not ideal for stenting, and chronic total occlusions of the LAD. Additionally, this approach may be considered for patients with extensive coronary artery disease who are of advanced age or have significant comorbidities, particularly when a heart team discussion supports hybrid coronary revascularization—combining LIMA grafting to the LAD with PCI for non-LAD lesions. For patients with a history of prior sternotomy, robotic-assisted CABG may be considered when PCI is not feasible and redo sternotomy CABG is deemed too invasive or high risk. This approach is feasible if the in situ IMA is available or if a composite graft can be constructed with inflow from an existing bypass graft, provided that direct anastomosis to the target vessel is achievable via mini-thoracotomy. However, robotic-assisted CABG has several contraindications. Absolute contraindications include previous pleural symphysis, extensive prior lung surgery, and prior use or injury of both IMAs. Relative contraindications consist of severe obesity, significant cardiac enlargement that reduces the available space between the heart and posterior chest wall, chest wall deformities such as severe pectus excavatum, and diffuse distal coronary disease that may limit revascularization feasibility. Hybrid PCI is generally performed after robotic-assisted CABG, most often during the same admission. However, in cases where a non-LAD lesion is the culprit in acute coronary syndrome and the LAD also has significant disease, PCI for the non-LAD lesion is performed first, followed by robotic-assisted CABG (reverse hybrid procedure). For cases involving a reverse hybrid procedure, if the LAD lesion is stable, the surgery is typically performed after a 4-week period of dual antiplatelet therapy.

### Statistical analysis

Continuous values are presented as median (interquartile range) or mean ± standard deviation, depending on the distribution of variables assessed by the Shapiro–Wilk test, unless otherwise noted. For variables with a normal distribution, the t-test was used to compare 2 groups, while for variables with a non-normal distribution, the Mann-Whitney U-test was used. Categorical values are reported as numbers (percentages), and the chi-squared test or Fisher’s exact test was used to compare 2 groups, as appropriate. Unless otherwise noted, definitions, terminology, and reported outcomes were consistent with the Society of Thoracic Surgeons (STS) database guideline. All p values were 2-sided, and a 5% level was considered significant. All analyses were conducted using the R software, version 4.2.3 (R Foundation for Statistical Computing, Vienna, Austria).

## Results

Out of a total of 1438 patients who underwent robotic-assisted CABG between January 2016 and December 2023, 12 patients underwent redo CABG. After excluding 11 patients with a history of non-CABG cardiac surgery, 1415 patients were included in the initial CABG group. The baseline characteristics of the patients are shown in **Table 1**. The mean age and STS-PROM of the entire cohort were 70 ± 11 years and 1.79 ± 2.86, respectively. Two patients in the redo group had end-stage renal disease on dialysis, and redo patients had higher rates of comorbidities, including chronic lung disease, cerebrovascular disease, peripheral artery disease, a history of prior PCI, and significantly higher STS-PROM compared with the initial CABG patients.

**Table 1:** Baseline characteristics

	Redo coronary artery bypass grafting		p value
	No (N = 1415)	Yes (N = 12)	
Age (years)	70 (63–77)	73 (68–81)	0.27
Female	334 (24)	5 (42)	0.26
Body mass index (kg/m^2^)	29 (25–32)	28 (25–29)	0.43
Society of Thoracic Surgeons score	0.90 (0.48–1.83)	7.05 (4.04–12.9)	**<0.001**
Hypertension	1256 (89)	12 (100)	0.38
Dyslipidemia	1182 (84)	11 (92)	0.70
Diabetes	557 (39)	6 (50)	0.56
Creatinine (mg/dL)	1.0 (0.9–1.2)	1.4 (0.9–1.8)	**0.03**
Dialysis	31 (2.2)	2 (17)	**0.03**
Chronic lung disease	228 (16)	5 (42)	**0.03**
Cerebrovascular disease	319 (23)	7 (58)	**0.008**
Peripheral artery disease	185 (13)	5 (42)	**0.01**
Prior myocardial infarction	676 (48)	8 (67)	0.31
Prior percutaneous coronary intervention	621 (44)	11 (92)	**0.002**
Atrial fibrillation	196 (14)	2 (17)	0.68
Left ventricular ejection fraction (%)	60 (53–65)	52 (36–61)	0.07
Number of diseased vessels			0.15
1	287 (20)	0	
2	495 (35)	4 (33)	
3	633 (45)	8 (66)	
Left main coronary artery disease	251 (17)	3 (25)	0.46
Left anterior descending artery disease	1330 (94)	10 (83)	0.16
Status			0.41
Elective	881 (62)	6 (50)	
Urgent	529 (37)	6 (50)	
Emergent	5 (0.4)	0	

Median (interquartile range) or n (%).

Bold: p <0.05.

Details of the robotic-assisted redo CABG are described in **Table 2**. Six patients (cases 1, 2, 4, 9, 11, and 12) underwent de novo IMA-LAD bypass; 4 patients (cases 3, 6, 7, and 8) underwent redo LAD bypass; and 2 patients (cases 5 and 10) underwent bypass for non-LAD PCI-ineligible or PCI-refractory lesions. Of the 10 patients who received LAD bypass, 4 patients underwent hybrid PCI to non-LAD lesions. Among the 4 patients who received redo LAD bypass, 2 patients (cases 3 and 6) had previously undergone LAD bypass with SVG, and the LIMA was used for the redo LAD bypass in these cases. One patient (case 7) had an occluded LIMA-LAD graft, and RIMA harvesting was attempted for a redo LAD bypass but was unsuccessful. As a result, a short segment of SVG was harvested and used for LAD bypass using the existing SVG to the diagonal branch as inflow. Another patient (case 8) had a stenosis at the LIMA anastomosis site with an otherwise healthy LIMA. The stenosed anastomosis was removed, the anastomosis site was enlarged, and a short segment of SVG was used as a graft between the LIMA and LAD. In case 10, the patient with end-stage renal disease undergoing hemodialysis, who presented with a non-ST-elevation myocardial infarction due to repeated in-stent restenosis in the ostial right coronary artery, underwent urgent surgery with robotic harvesting of the RIMA from the right side, followed by direct anastomosis of the RIMA to the right coronary artery via a right mini-thoracotomy.

**Table 2:** Details of patients undergoing robotic-assisted redo coronary artery bypass grafting

Case	Age (years)/sex	Previous procedure	Presentation	Coronary lesions	Procedure	Follow-up
1	70, M	RIMA-RCA	Angina	Proximal-mid LAD 90%; proximal LCX 90%	LIMA-LAD; hybrid PCI to LCX	2.0 years Alive
2	75, F	SVG-OM1-OM2; SVG-RCA	HFpEF on IABP	LMCA 99%	LIMA-LAD	2.4 years Alive
3	58, M	SVG-LAD; SVG-Dx; SVG-RPDA	Angina	Occluded LAD with 90% LIMA graft stenosis; RCA occlusion with RPDA graft occlusion	LIMA-LAD	2.5 years Alive
4	58, M	RIMA-OM; SVG-RPDA	NSTEMI on IABP	Proximal LAD in-stent restenosis	LIMA-LAD	3.1 years Alive
5	84, F	SVG-RCA; aortic valve replacement	Angina	Ostial LCX 95%	LIMA-OM	1.4 years Dead (age)
6	82, M	SVG-LAD; SVG-OM; SVG-RPDA	NSTEMI	LMCA 99%; occluded SVG to LAD; occluded SVG to OM	LIMA-LAD; hybrid PCI to LMCA-LCX	5.6 years Alive
7	64, M	LIMA-LAD; SVG-Dx	Angina	Ostial LAD occlusion with occluded LIMA; mid LCX 80%; occluded RCA	Attempted RIMA harvest; LAD bypass with de novo SVG with inflow from SVG to Dx	5.7 years Alive
8	88, M	LIMA-LAD; SVG-Dx; SVG-OM1; RIMA-RCA	Angina	Occluded LAD with 90% LIMA anastomosis stenosis; occluded SVG to Dx; stenosed SVG to OM1	LIMA-SVG-LAD; hybrid PCI to LCX	3.7 years Dead (age)
9	69, M	LIMA-Dx; SVG-OM; radial-RPDA	HFrEF	Proximal LAD in-stent restenosis	RIMA-LAD	6.0 years Alive
10	69, F	LIMA-LAD	NSTEMI	Ostial RCA in-stent restenosis	RIMA-RCA	1.7 years Dead (infection)
11	81, F	SVG-RCA; mitral valve repair	NSTEMI on IABP	Ostial LMCA 90%; proximal LCX 90%	LIMA-LAD; hybrid PCI to LCX	3 months Dead (heart failure)
12	79, F	SVG-D1; radial-D2; SVG-ramus-OM	NSTEMI; HFrEF	Occluded LAD; occluded SVG graft to D1	LIMA-LAD	4.5 years Dead (infection)

D/Dx: diagonal; HFpEF: heart failure with preserved ejection fraction; HFrEF: heart failure with reduced ejection fraction; IABP: intra-aortic balloon pump; LAD; left anterior descending artery; LCX; left circumflex artery; LIMA: left internal mammary artery; LMCA: left main coronary artery; NSTEMI: non-ST-elevation myocardial infarction; OM: obtuse marginal artery; PCI: percutaneous coronary intervention; RCA: right coronary artery; RIMA: right internal mammary artery; RPDA: right posterior descending artery; SVG: saphenous vein graft.

**Table 3** shows the operative characteristics and early outcomes. While operating room time and postoperative lengths of stay were significantly longer, and the need for blood transfusion was significantly higher in the redo group compared to the initial group, there were no conversions to sternotomy, stroke, bleeding, unplanned revascularization, or in-hospital mortality among the redo patients.

**Table 3:** Operative characteristics and early outcomes

	Prior cardiac surgery		p value
	No (N = 1415)	Yes (N = 12)	
Internal mammary artery use	1412 (>99)	10 (83)	**<0.001**
Number of bypass grafting			0.66
1	1288 (91)	12 (100)	
2	111 (7.8)	0	
3+	16 (1.1)	0	
Operating room time (hours)	5.4 (4.9–6.0)	7.4 (5.3–8.2)	**<0.001**
Extubation in the operating room	1270 (90)	9 (75)	0.12
Conversion to sternotomy	23 (1.6)	0	>0.99
Hybrid percutaneous coronary intervention	622 (44)	4 (33)	0.66
Complete revascularization	882 (62)	6 (50)	0.39
Ventilation >24 hours	20 (1.4)	1 (8.3)	0.16
Intensive care unit length of stay (hours)	32 (25–55)	45 (22–58)	0.73
Stroke	12 (0.8)	0	>0.99
Bleeding	17 (1.2)	0	>0.99
Unplanned revascularization	16 (1.1)	0	>0.99
Renal failure	35 (2.5)	0	>0.99
New-onset atrial fibrillation	259 (18)	1 (8.3)	0.71
Deep wound infection	1 (<0.01)	0	>0.99
Intra- and postoperative blood transfusion	208 (15)	5 (42)	**0.02**
Postoperative length of stay (days)	4.0 (3.0–5.0)	5.5 (4.0–7.3)	**0.02**
In-hospital mortality	14 (1.0)	0	>0.99

Median (interquartile range) or n (%).

Bold: p <0.05.

The follow-up results for the redo patients are shown in **Table 2** (last column). One patient, an 81-year-old frail woman with left main disease who presented with a non-ST-elevation myocardial infarction and requiring an intra-aortic balloon pump and urgent surgery (case 11), died within 1 year of surgery. This resulted in a 1-year survival rate of 92%. No patient experienced a stroke or myocardial infarction during follow-up.

## Discussion

Although several studies have shown satisfactory results for minimally invasive cardiac surgery in patients with a history of prior sternotomy, including redo CABG, ^17–19)^ the experience with robotic-assisted redo CABG has been limited.^20)^ In this study, we demonstrated 12 cases of robotic-assisted CABG in patients with a history of CABG, showing promising early outcomes. A longer operating room time in redo patients compared to initial CABG cases is reasonable due to several factors, including extensive adhesions involving the lungs and surrounding tissues requiring careful dissection, prolonged LIMA harvesting due to previous surgical adhesions, and additional time needed to identify the prior bypass site and determine an appropriate anastomosis location.

Currently, the optimal strategies for repeat coronary revascularization remain controversial. In general, redo CABG is associated with higher rates of in-hospital mortality and stroke compared to PCI in patients with prior CABG.^21,22)^ Specifically, these studies reported that 30-day mortality after redo CABG ranged from 2.8% to 22%. Therefore, PCI to a native vessel or SVG is often a more favorable option than redo CABG, especially if LIMA-LAD bypass is not planned or if the patient already has a patent LIMA-LAD. On the other hand, when PCI is not feasible, a patent IMA-LAD is not present, or an IMA is available for LAD bypass, CABG is often chosen as the revascularization strategy for patients with refractory angina who have an acceptable risk of reoperation.^6)^ In addition, studies have shown that redo CABG is preferred to PCI in patients with fewer functional grafts, more chronic total occlusions, and lower systolic function. LIMA-LAD bypass may also confer a long-term survival advantage.^22,23)^

Robotic-assisted redo CABG offers LIMA-LAD bypass with a less invasive approach compared to traditional redo sternotomy CABG. This technique potentially allows for the expansion of indications to include patients with coronary lesions not amenable to PCI and at high risk for redo sternotomy CABG, or provides superior outcomes with LIMA-LAD bypass compared to PCI in patients with prior CABG and unprotected left main or complex LAD lesions while avoiding the risks associated with redo sternotomy. Patients with prior CABG and PCI-ineligible or PCI-refractory non-LAD lesions, who are typically not favorable candidates for redo sternotomy CABG, may also benefit from this less invasive approach. Ideal indications should include de novo LIMA-LAD bypass in patients with a history of combined CABG of non-LAD lesions with other cardiac procedures (e.g., aortic valve replacement and right coronary artery bypass with a non-LIMA graft) or patients with a history of unsuccessful PCI for acute coronary syndrome requiring CABG to the non-LAD lesion when accessible via mini-thoracotomy. However, even in patients with LAD bypass graft failure, this approach with redo LAD bypass was feasible in some cases, as demonstrated in this study. Although our small cohort cannot conclude the efficacy of this procedure over the conventional approach, it has the potential to serve as an alternative or contemporary option to PCI and redo sternotomy CABG in appropriately selected patients. For successful robotic-assisted redo CABG, a thorough evaluation beyond the general redo CABG assessment is essential. This includes a careful assessment of the feasibility of robotic IMA harvesting, the accessibility of the target bypass site via thoracotomy, and the assurance of inflow when in situ IMA grafts are not available, as demonstrated in case 7 of this study. Computed tomography and other imaging modalities should be utilized to assess anatomical feasibility and anticipate potential adhesions. Given the risk of severe adhesions in redo cases, careful port insertion is crucial to prevent cardiac injury, while meticulous dissection is necessary to avoid IMA injury during harvesting. In this context, 3-dimensional visual enhancement may provide an advantage by improving spatial awareness and precision during dissection. Further experience and investigation are needed to identify appropriate candidates and to determine the efficacy of this approach in terms of both early and long-term outcomes compared with the conventional approach.

This study has several important limitations. First and foremost, it is a single-center retrospective study with a small number of patients who underwent robotic-assisted redo CABG. The comparison between the initial and redo CABG patients was intended only to highlight the heterogeneity of baseline and operative characteristics between the groups. Additionally, a direct comparison between robotic-assisted redo CABG and conventional redo CABG via sternotomy was not performed due to differences in anatomical severity and patient characteristics, which made a meaningful comparison challenging. Instead, we focused on evaluating the feasibility of robotic-assisted redo CABG by comparing it with initial robotic-assisted CABG. Robotic-assisted CABG was performed in highly selected patients, which raises concerns regarding selection bias and its potential impact on clinical outcomes and the generalizability of the results. Additionally, outcomes may have been influenced by the surgical team’s experience and the inherent learning curve associated with robotic-assisted CABG. Finally, the database lacks detailed procedural data for the initial CABG. Despite these limitations, our experience with this uncommon procedure will provide valuable insights, contributing to the expansion of real-world knowledge and application.

## Conclusion

We demonstrated that robotic-assisted redo CABG is a safe and effective procedure with satisfactory early outcomes. This approach may serve as a viable alternative or contemporary option to PCI and sternotomy redo CABG in appropriately selected patients.

## Declarations

### Ethics approval and consent to participate

The study protocol was approved by our Institutional Review Board (IRB 45CFR164.512). Individual patient consent was waived due to the retrospective nature of the study.

### Consent for publication

Not applicable.

### Funding

This research was funded by the Sharpe-Strumia Foundation of Bryn Mawr Hospital.

### Data availability

The data that support the findings of this study are available from the corresponding author upon reasonable request.

### Authors’ contributions

Conceptualization: FPS; Data curation: YY, SS, MACW; Formal analysis: YY; Investigation: GT, DDS, BR, FPS; Methodology: YY, SS; Supervision: GT, DDS, BR, FPS; Visualization: YY; Writing original draft: YY; Writing review and editing: SS, GT; All authors have read and approved the final version of the manuscript.

### Disclosure statement

Francis P. Sutter is on the Speakers Bureau for Intuitive Surgical, Inc. The remaining authors declare that they have no conflicts of interest.
